# Postoperative Ileus after Stimulation with Probiotics before Ileostomy Closure

**DOI:** 10.3390/nu13020626

**Published:** 2021-02-15

**Authors:** Ángela Rodríguez-Padilla, Germán Morales-Martín, Rocío Pérez-Quintero, Juan Gómez-Salgado, Rafael Balongo-García, Carlos Ruiz-Frutos

**Affiliations:** 1 Department of General Surgery, Infanta Elena University Clinical Hospital, 21080 Huelva, Spain; angela.rodriguez.padilla@gmail.com (Á.R.-P.); german.dr@hotmail.com (G.M.-M.); 2 Department of General Surgery, Juan Ramón Jiménez University Clinical Hospital, 21005 Huelva, Spain; roc14589@hotmail.com; 3 Department of Sociology, Social Work and Public Health, Faculty of Labour Sciences, University of Huelva, 21007 Huelva, Spain; frutos@uhu.es; 4 Safety and Health Postgraduate Programme, Universidad Espíritu Santo, Guayaquil 092301, Ecuador; 5 Chief of Gastrointestinal Surgery, Department of General Surgery, Juan Ramón Jiménez University Clinical Hospital, 21005 Huelva, Spain; rafael.balongo.sspa@juntadeandalucia.es

**Keywords:** probiotics, postoperative ileus, efferent loop stimulation, ileostomy closure, protective ileostomy

## Abstract

Loop ileostomy closure after colorectal surgery is often associated with Postoperative ileus, with an incidence between 13–20%. The aim of this study is to evaluate the efficacy and safety of preoperative stimulation of the efferent loop with probiotics prior to ileostomy closure in patients operated on for colorectal carcinoma. For this, a prospective, randomized, double-blind, controlled study is designed. All patients who underwent surgery for colorectal carcinoma with loop ileostomy were included. Randomized and divided into two groups, 34 cases and 35 controls were included in the study. Postoperative ileus, the need for nasogastric tube insertion, the time required to begin tolerating a diet, restoration of bowel function, and duration of hospital stay were evaluated. The incidence of Postoperative ileus was similar in both groups, 9/34 patients stimulated with probiotics and 10/35 in the control group (CG) with a *p* = 0.192. The comparative analysis showed a direct relationship between Postoperative ileus after oncological surgery and Postoperative ileus after reconstruction surgery, independently of stimulation. Postoperative ileus after closure ileostomy is independent of stimulation of the ileostomy with probiotics through the efferent loop. There seem to be a relationship between Postoperative ileus after reconstruction and the previous existence of Postoperative ileus after colorectal cancer surgery.

## 1. Introduction

The performance of a temporary ileostomy in patients operated on for colorectal carcinoma, by means of an anterior resection with total mesorectal excision, reduces the morbidity associated with anastomotic leak [1]. However, reconstructive surgery is not risk-free. The incidence of complications following ileostomy closure, such as diversion colitis, small bowel obstruction, surgical wound infections, postoperative ileus, anastomotic leak, fistula, perforation, abscess, bleeding, or hernia [2,3], ranges from 18% to 40%. The most common complication is postoperative ileus, with an incidence of 13–20% [4]. As a result, this can lead to a significant socioeconomic impact, the worsening of a patients’ quality of life due to greater discomfort, increased risk of nosocomial infections and morbidity associated with surgery, and an increase in postoperative mortality; all of this conditioning a prolonged hospital stay and higher healthcare costs [5].

On the other hand, previous studies have shown that the colon defunctionalization produces dysbacteriosis and atrophy of its villi and muscular layers in the excluded colon segment, as well as a loss of segmentary contractility that could produce a reduction in the ability for absorption [6,7]. To avoid this, two lines of studies have been proposed; early closure of the loop ileostomy, which would be the best option [8], or late closure after stimulation of the efferent loop. There are many publications on stimulation of the efferent loop prior to stoma closure. Different protocols exist, with different substances administered, from autologous fecal fluid to physiological serum with thickeners, passing through short-chain fatty acids, liquid diet, and sucrose [9].

Based on the need to return the excluded intestine to its function, pre-enabling it before surgery reconstruction, and demonstrated bacterial dysbiosis, we decided to administer probiotics through the efferent loop. Probiotics are increasingly being applied in gastrointestinal pathology. They are non-pathogenic live bacteria, such as *Lactobacillus*, *Bifidobacterium*, and *Enterococcus*, which present antimicrobial and immunomodulatory activity and improve the intestinal barrier. Supplied in adequate amounts, they promote health benefits on the host [10].

## 2. Materials and Methods

### 2.1. Study Design

We conducted a prospective, randomized, multicenter, double-blind experimental study, comparing two groups of patients operated on for colorectal carcinoma with loop ileostomy. The CONSORT statement criteria were followed.One group included patients treated with stimulation of the efferent loop with probiotics prior to transit reconstruction surgery; the other control group was stimulated without giving any substance.

### 2.2. Simple Size

The sample size was calculated according to the incidence of postoperative complications (PI) obtained after ileostomy closure published in a recent systematic review [4]. The assumed reduction in PI was 50% (20–10%). With a loss adjustment of 15%, it was necessary to have 30 patients per group. We recruited 34 patients in the stimulated group and 35 patients in the control group for a statistical level of 95% and a power of 0.8.

### 2.3. Selection of Patients

**Figure 1:**
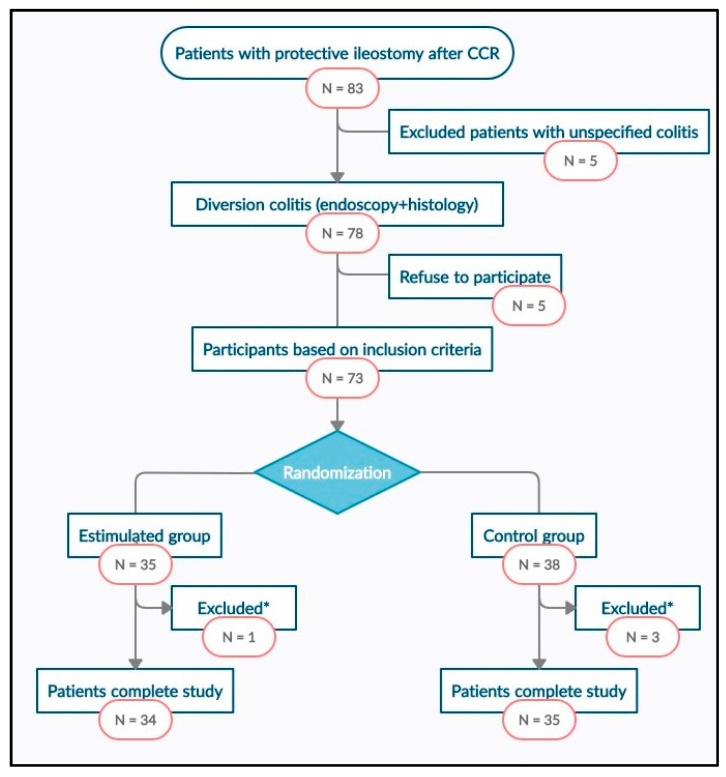
Study flowchart. CRC: Colorectal Cancer. *: excluded patients with an anastomotic leak.

Between January 2017 and December 2018, all the patients from the three participating centers included in the surgical waiting list for temporary stoma closure after colorectal carcinoma were consecutively evaluated to determine their inclusion in the study. The selection flowchart of the study patients can be seen in Figure 1.

The inclusion criteria were being over 18 years of age, having protective ileostomy after colorectal carcinoma surgery free of disease, with endoscopic and histological confirmation of diversion colitis, and having signed the informed consent. 

The exclusion criteria were being under 18 years of age, clinical history, and histological confirmation of inflammatory bowel disease with colorectal involvement and refusal to participate in the study. Abandonment criteria were loss during follow-up, exitus, and anastomotic dehiscence after reconstruction surgery.

### 2.4. Randomization and Intervention

After excluding patients who did not meet the criteria, randomization was performed by using a computer-generated sequence with the statistical software EPIDAT 4.1 (version 4.1. Servizo de Epidemioloxía da Dirección Xeral de Saúde Pública da Consellería de Sanidade (Xunta de Galicia), Santiago de Compostela, A Coruña, España). Two groups were designed:

- Stimulation group (SG): preoperative stimulation of the distal limb of the ileostomy loop with probiotics was performed during the 20 days prior to surgery every second day. During the process and after it, the patient himself registers the appearance of symptoms after each stimulation session: abdominal pain, emission of gas and stool. A sterile Foley catheter No.14 Ch connected to an infusion set was introduced through the defunctioned bowel to allow the slow infusion, for 20–30 min, of a solution with 4.5 mg of probiotics diluted in 250 mL of 0.9% physiological saline. Each preparation was made under sterile conditions and maintaining the cold chain. Vivomixx^®^ lyophilized live bacteria, count by SIGMA-TAU PHARMACEUTICALS and manufactured in Spain by GRIFOLS S.A., contained 4.5 × 10^11^ of live bacteria in each preparation:
  ○ Four strains of *Lactobacillus*:
    ■ Lactobacillus acidophilus DSM 24735
    ■ Lactobacillus plantarum DSM 24730
    ■ Lactobacillus paracasei DSM 24733
    ■ *Lactobacillus delbrueckii* subsp. bulgaricus DSM 24734.
  ○ Three strains of *Bifidobacterium*:
    ■ Bifidobacterium breve DSM 24732
    ■ Bifidobacterium longum DSM 24736
    ■ Bifidobacterium infantis DSM 24737
  ○ One strain of *Streptococcus*
    ■ Streptococcus thermophilus DSM 24731.
- Control group (CG): exactly the same procedure was carried out, but with the infusion set closed. During the process and after it, the patient himself registers the appearance of symptoms after each stimulation session: abdominal pain, emission of gas and stool.

After 10 stimulation sessions, reconstructive surgery was performed within the following 48 h.

### 2.5. Surgery and Follow-Up

All patients were admitted to the hospital the day before surgery, fasting, receiving antithrombotic prophylaxis (enoxaparin 40 mg subcutaneous), and premedication according to the pre-anesthesia instruction sheet. The reconstruction surgery was carried out by three expert surgeons from the Colorectal Surgery department. A parastomal incision was made and carried out sharply into the peritoneal cavity. The anastomosis was lateral-lateral, either manual or mechanical, according to the decision of the surgeon. Every surgeon was allowed to decide as to whether to change to a median laparotomy procedure. Complications or events that happened during surgery were recorded in the surgical procedure protocol. General anesthesia was given to all patients and, after extubation and stabilization in the postoperative resuscitation room, they went directly to the hospitalization ward.

Follow-up during hospitalization was carried out by the team of the Colorectal Surgery department of each center, recording any postoperative complications, with special vigilance of abdominal pain, the passage of flatus or stool with correct quantification and initiation of oral tolerance, postoperative ileus, and the need of nasogastric tube insertion. Patients were discharged from the hospital after re-establishing intestinal transit, adequate oral tolerance, and stool control, and recording the length of stay in the hospital.

### 2.6. Masking

To ensure masking of the patients, all underwent the same diagnostic procedure. During the stimulation sessions, both the solution with probiotics and the serum system was covered by an opaque protective envelope, which prevented observing the color and transparency of the fluid, or whether the system was open or closed. The stimulation sessions were performed by a single surgeon, who was also in charge of preparing the dilution.

The endoscopist, the pathologist, and the surgeon who performed the surgical intervention and the follow-up, as well as surgeons who participated, after the surgery, in the hospitalization process, did not know whether the patient had received probiotics or not.

### 2.7. Assessment Criteria

The main endpoint was the effect caused by stimulation of the efferent loop with probiotics on the appearance of postoperative ileus, the need for nasogastric tube insertion, the time required to begin tolerating a diet, restoration of bowel function, and the duration of hospital stay when compared with CG after surgery. Postoperative ileus was defined as impaired gastrointestinal motility leading to a delayed return of bowel function, measured using the validated I-FEED scale as: intolerance to oral intake or interruption of oral diet for more than 72 h, absence of bowel sounds, abdominal distention, or need for inserting a nasogastric tube [11,12].

Secondary evaluation criteria were the duration of hospital stay, measured as days of admission, and the appearance of postoperative ileus after colorectal surgery with a protective ileostomy, measured by the evolutions recorded during the first surgery, also applying the I-FEED scale (Intake, Feeling nauseated, Emesis, physical Exam, and Duration of symptoms).

### 2.8. Statistical Analysis 

A descriptive univariate analysis of sociodemographic and clinical variables was performed. The Kolmogorov-Smirnov test was used to verify the normality of the quantitative variables. To describe the quantitative variables, the mean and standard deviation were used, and the median and interquartile range for those variables that did not follow a normal distribution. For qualitative variables, frequencies and percentages were used. Afterward, to verify the main objectives, a bivariate analysis was performed. A contrast test of proportions based on the Chi-square test was used in order to determine whether stimulation of the efferent loop with probiotics prior to the closure of the protective ileostomy reduces the appearance of postoperative ileus, the need for nasogastric tube insertion, the time required to begin tolerating a diet, restoration of bowel function and the possibility of reducing the duration of hospital stay. For the correlation of quantitative variables, Spearman’s rank correlation coefficient was used. In all cases, a statistical significance of 5% was required. Statistical analyses were performed using the statistical program SPSS version 24.0 (IBM: Armonk, NY, USA), with the support of calculation tools provided by the software Microsoft Excel or R (Microsoft: Redmond, WA, USA).

### 2.9. Ethical Aspects

The project was performed with the consent of the Ethics Coordinating Committee for Biomedical Research of Andalusia, Spain, and registered with the project number 2017/331191354. Written informed consent was requested to participate in the study, giving details of both the study objectives and the methodology to be followed. The data was kept anonymous, maintaining the confidentiality and anonymity of the participants. The study was conducted under the “Ethical Principles for Medical Research Involving Humans” contained in the latest version of the Helsinki Declaration (Fortress Amendment, Brazil, October 2013). 

## 3. Results

### 3.1. Study Population

Between January 2017 and December 2018, 83 disease-free patients with protective ileostomy after colorectal carcinoma resection were reviewed and included in the surgical waiting list for intestinal transit reconstruction. Seventy-eight of them met the endoscopic and histological criteria for diversion colitis diagnosis, and 73 patients were finally randomized into two groups, intervention (*n* = 35) and control (*n* = 38). Sixty-nine patients completed the study, one of them from SG and three from the CG abandoning the study because of anastomotic fistula. The study flowchart is presented in Figure 1. There were no significant differences between SG and CG in terms of sociodemographic, clinical, or surgical variables (Table 1).

Abdominal pain was the only symptom observed during stimulation, which appeared in 20.5% of patients SG (*n* = 7) compared to 14.3% CG (*n* = 5). There were no statistically significant differences between the two groups. Abdominal pain was evaluated using the visual analog scale (VAS). Pain was moderated in SG, only present in the first stimulation sessions and disappearing afterward. Pain was mild-moderate in CG in all stimulation sessions and disappeared after their completion.

### 3.2. Main Assessment Criteria

The results of the main endpoint and postoperative evolution are described in Table 2. The incidence of postoperative ileus was similar in both groups, present in 10/34 (29.4%) SG and 11/35 (31.4%) CG with *p* = 0.192. There were no significant differences regarding the need for a nasogastric tube, the time required to begin tolerating a diet, restoration of bowel function, and the duration of hospital stay. After closure ileostomy, SG showed initiation of oral tolerance 24-h earlier than CG (2 versus 3 days respectively), which is considered statistically non-significant. The passage of gases and restoration of bowel function appeared 2 days after surgery in both groups. SG had an interval between 1–20 days to the emission of gases and 1–21 days to the restoration of bowel function compared to CG, which had an interval of 1–48 days and 1–48 days, respectively. The hospital stay was also shorter in SG but without statistical significance. In SG, nine patients needed a nasogastric tube (26%) to manage postoperative ileus. In CG, all the patients who presented postoperative ileus required a nasogastric tube as part of their treatment (31.4%).

### 3.3. Secondary Assessment Criteria: Postoperative Ileus and Colorectal Surgery

**Figure 2:**
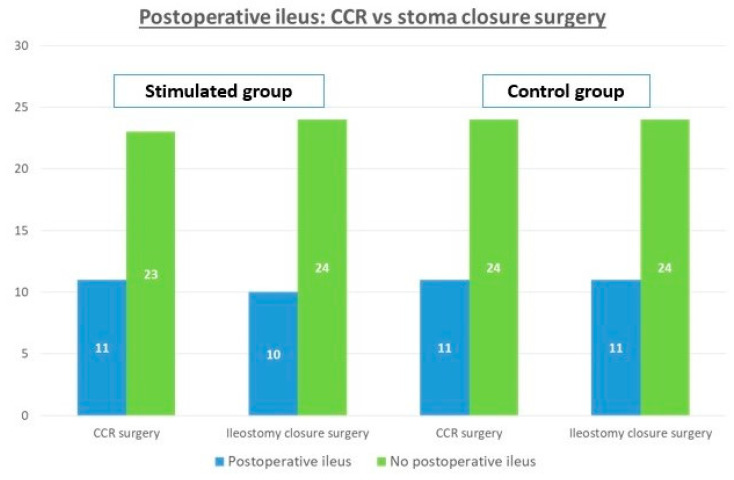
Comparison of postoperative ileus and the need for a nasogastric tube (NT) in colorectal surgery (CCR) and after reconstructive surgery.

From the comparative analysis, the incidence of postoperative ileus after closure ileostomy was found to be similar to the one that occurred in primary colorectal surgery. In other words, patients who presented ileus after reconstruction surgery also presented it in the previous colorectal surgery. In the SG, postoperative ileus after colorectal surgery happened in 11/23 patients, and after reconstruction, it appeared in 10 of these 11 cases. In the same way, in the CG postoperative ileus after colorectal surgery happened in 11/24 patients, and after reconstruction, the same 11 patients presented it. Therefore, there is a direct relationship between ileus after colorectal surgery and after intestinal transit reconstruction, independent of stimulation with *p* < 0.001 (Figure 2).

## 4. Discussion

The appearance of postoperative ileus after closure ileostomy has been analyzed in several studies [4,5,8,13,14]. Garfinkle et al. published the first systematic review in 2019 [4]. It includes 9528 patients in 67 studies, most of them retrospective record. The authors point out that its incidence depends on the definition that we use about ileus and that these variations cause great heterogeneity in the included studies, so we will need to continue investigating and defining it with homogeneous criteria. In fact, more than half of the studies in this meta-analysis (39/67) did not have defined criteria for postoperative ileus (studies with a lower incidence), and among those, there is no coincidence in the definition.

In order to find a common denominator to compare different studies, we relied on the study published by the ASER (American Society of Enhanced Recovery) and POQI (Perioperative Quality Initiative) working group [11], which establishes definitions based on the Symptom score for postoperative gastrointestinal dysfunction (using terms such as ileus, paralytic ileus, postoperative ileus, or prolonged ileus) including oral tolerance, nausea, vomiting, bloating, and duration of these symptoms. This validated scoring system called I-FEED [12] was used in our study because, in our opinion, it appears to be the most objective and extrapolated way to determine the appearance of postoperative ileus, defined as postoperative gastrointestinal dysfunction, which is different from postoperative gastrointestinal intolerance that is resolved in 24–48 h, and allows it to be compared with other studies in the future.

The idea of the stimulation of the efferent loop prior to reconstructive surgery was born after raising the hypothesis that the appearance of postoperative ileus is due to the structural and functional changes that take place in the dysfunctional intestine, such as muscle and villous atrophy or decreased absorption capacity [7,14]. Distal bowel and colon, which are unready to re-establish transit, cause intestinal paralysis. In this regard, several articles have been published [13,14,15,16,17], but the first systematic review was published by Rombey et al. [9] in 2019, including eight studies with 267 patients. Despite the excellent initial results, there is not sufficient evidence to recommend the routine implementation of preoperative bowel stimulation in clinical practice. This is due to the heterogeneity of the studies, which have not enough statistical significance, and whose results are difficult to compare with each other since some compare patients undergoing proctocolectomy with ileoanal J anastomosis and other patients with low anterior resection. Only one study was randomized, three were unique clinical cases, and there was diversity in terms of the substance used during stimulation and its duration. Only two comparative studies found that preoperative bowel stimulation reduced the time to restoration of bowel function when compared to standard preoperative care [14,16]. The reduction was statistically significant with regards to the mean time to the tolerance of solid food. The mean time to passing flatus or stool was significantly lower, but the mean time to tolerance of liquids did not significantly differ between the intervention and control groups in the two studies reporting this outcome. The case reports results matched the comparative studies results [15,17]. Preoperative bowel stimulation significantly reduced the mean postoperative ileus in two comparative studies [14,16], by 2.1 and by 2.6 days, respectively. In the two case reports [15,17], the postoperative ileus was of two and four days, respectively, which is consistent with the RCT result [14].

The hypothesis of the present study was motivated by the absence of clear scientific evidence on the benefit of stimulation of the efferent loop. We used a double-blind design, maintained until the end of the study, a standardized definition of postoperative gastrointestinal dysfunction, which had not been used up to now, and based our trial on comparative studies and published meta-analyzes on intestinal pathophysiological changes caused by the loss of intestinal flora in the excluded colon segment [6,7,8,9,14]. We decided to carry out our study to compare results after stimulation of the efferent loop with probiotics in patients previously operated on for colorectal carcinoma. Based on systematic reviews about the use of probiotics as treatment of other gastrointestinal pathologies [18,19,20], we considered that this stimulation would achieve the repopulation of the excluded colon, activating the cellular absorption mechanisms, increasing motility, and decreasing efferent loop atrophy, so that after closure ileostomy, the return to normality would be faster, with a shorter time required to re-establish oral transit and tolerance, reducing the onset of postoperative ileus and the duration of hospital stay. We chose the formulation of probiotics with the highest number of strains, which had an adequate synergistic and complementary effect between them, and the highest concentration. Another positive point is the absence of allergens such as gluten, lactose, or soy.

In our study, after the inclusion and randomization of 73 patients, no statistically significant differences were detected in terms of sociodemographic, clinical, or surgical variables. A notable reduction in surgical time was observed in the SG, which we associate with the thickening of the efferent loop, resulting from the stimulation, and which facilitates the performance of anastomosis. We also did not find differences between the incidence of paralytic ileus in the SG (*n* = 10; 29.4%) and the CG (*n* = 11; 31.4%), with a slight increase in it, compared to studies collected in systematic reviews published by Rombey and Garfinkle [4,9], that we associate to the standardization of the definition of postoperative gastrointestinal dysfunction. The difference in terms of duration of hospital stay was 1 day, with a median of 4 days in the SG and 5 days in the CG. This was associated with a 24-h difference in oral tolerance in the SG, in which 58% of patients tolerated a liquid diet in the first 24–48 h, compared to the CG, which took 48–72 h. These results are similar to those reported by other studies [9,14,15]. Moreover, it was observed that the incidence of postoperative ileus was exactly the same as that presented in colorectal surgery and that this was due to the fact that all the patients who presented ileus after reconstruction surgery also did in the previous colorectal surgery. This fact is important, as the closuring of ileostomy does not present the traditional risk factors of colorectal surgery, such as surgical time, increased level of fluids, and loss of blood, among others [4,21,22,23].

## 5. Conclusions

Therefore, given the results obtained in our study, we have not been able to demonstrate the benefit of stimulating the efferent loop with probiotics on the appearance of postoperative ileus. Scientific evidence is insufficient to incorporate this procedure into our protocol prior to closure ileostomy. We will need more studies to establish its impact on operating results. We have indeed observed a direct relationship between the onset of ileus after reconstruction surgery and the fact that these patients had previously presented the same affection after colorectal carcinoma surgery, a finding that may represent a new line of research.

## Figures and Tables

**Table 1:** Demographics, clinics and surgical characteristics.

	Stimulated Group (*n* = 34)	No Stimulated Group (*n* = 35)	*p*
Demographics			
Age (years)	65 (45–81)	68 (41–80)	0.130
Sex ratio (Male:Female)	23:11	25:10	0.733
BMI (kg/m^2^)	23.5 (21.6–32.6)	27.6 (18.8–40.2)	0.091
ASA			0.483
ASA I-II	31	30	
ASA III	3	5	
Smoker/nonsmoker	20/14	23/12	0.826
Colorectal surgery			
Surgical procedure:			0.129
LAR	25	25	
uLAR	9	10	
Surgical approach:			0.551
Laparoscopic	24	24	
Open	10	11	
Type of anastomosis:			0.430
Stapled EEA	31	33	
Coloanal anastomosis	3	2	
Neoadjuvant therapy	26	25	0.239
Adjuvant treatment	26	28	0.256
Ileostomy closure			
Time between surgeries (months)	12 (8–37)	9 (6–32)	0.813
Surgery:			0.690
Small bowel resection	33	34	
Ileocecal resection	1	1	
Surgical approach:			0.291
Peri-ileostomy	30	28	
Midline laparotomy	4	7	
Type of anastomosis:			0.355
Sewn	29	29	
Stapled	5	6	
Time (minutes)	50 (30–70)	65 (50–120)	0.053

BMI: body mass index. ASA: American Society of Anesthesiologists Classification. LAR/uLAR: low anterior resection/ultralow anterior resection. EEA: end to end anastomosis stapler.

**Table 2:** Postoperative results.

	Stimulated Group (*n* = 34)	No Stimulated Group (*n* = 35)	*p*
Postoperative ileus, *n* (%)	10 (29.4%)	11 (31.4%)	0.192
Nasogastric tube, *n* (%)	9 (26%)	11 (31.4%)	0.116
Time to tolerating a diet, days-mean (range)	2 (1–24)	3 (2–50)	0.619
Start of the passage of flatus, days-mean (range)	2 (1–20)	2 (1–48)	0.173
Start of the passage of stool, days-mean (range)	3 (1–21)	3 (1–48)	0.184
Postoperative stay, days-mean (range)	4 (4–26)	5 (4–56)	0.105

The median has been used as a measure of central tendency for the evaluation of variables as the initiation of oral tolerance, gas emission, restoration of transit, and the duration of hospital stay.

## Data Availability

All data generated or analyzed during this study are included in this published article.
